# The Characteristics of Patients With Possible Transient Ischemic Attack and Minor Stroke in the Hunter and Manning Valley Regions, Australia (the INSIST Study)

**DOI:** 10.3389/fneur.2020.00383

**Published:** 2020-05-15

**Authors:** Shinya Tomari, Parker Magin, Daniel Lasserson, Debbie Quain, Jose M. Valderas, Helen M. Dewey, P. Alan Barber, Neil J. Spratt, Dominique A. Cadilhac, Valery L. Feigin, Peter M. Rothwell, Hossein Zareie, Carlos Garcia-Esperon, Andrew Davey, Nashwa Najib, Milton Sales, Christopher R. Levi

**Affiliations:** ^1^Priority Research Centre for Stroke, University of Newcastle and Hunter Medical Research Institute, Newcastle, NSW, Australia; ^2^Discipline of General Practice, University of Newcastle, Newcastle, NSW, Australia; ^3^Institute of Applied Health Research, University of Birmingham, Birmingham, United Kingdom; ^4^Department of Neurology, John Hunter Hospital, Newcastle, NSW, Australia; ^5^Health Service & Policy Research Group, University of Exeter – Saint Lukes Campus, Exeter, United Kingdom; ^6^Faculty of Medicine, Nursing and Health Sciences, Box Hill Hospital, Monash University, Melbourne, VIC, Australia; ^7^Department of Medicine, University of Auckland, Auckland, New Zealand; ^8^Stroke and Aging Research, School of Clinical Sciences at Monash Health, Monash University, Clayton, VIC, Australia; ^9^Florey Institute of Neuroscience and Mental Health, University of Melbourne, Melbourne, VIC, Australia; ^10^AUT University, National Institute for Stroke & Applied Neurosciences, Auckland, New Zealand; ^11^Nuffield Department of Clinical Neuroscience, Centre for Prevention of Stroke and Dementia, University of Oxford, Oxford, United Kingdom; ^12^The Ingham Institute, SPHERE, Sydney, NSW, Australia

**Keywords:** transient ischemic attack, minor stroke, stroke-mimic syndrome, atrial fibrillation, anticoagulation therapy

## Abstract

**Background:** Transient ischemic attack (TIA) and minor stroke (TIAMS) are risk factors for stroke recurrence. Some TIAMS may be preventable by appropriate primary prevention. We aimed to recruit “possible-TIAMS” patients in the INternational comparison of Systems of care and patient outcomes In minor Stroke and TIA (INSIST) study.

**Methods:** A prospective inception cohort study performed across 16 Hunter–Manning region, Australia, general practices in the catchment of one secondary-care acute neurovascular clinic. Possible-TIAMS patients were recruited from August 2012 to August 2016. We describe the baseline demographics, risk factors and pre-event medications of participating patients.

**Results:** There were 613 participants (mean age; 69 ± 12 years, 335 women), and 604 (99%) were Caucasian. Hypertension was the most common risk factor (69%) followed by hyperlipidemia (52%), diabetes mellitus (17%), atrial fibrillation (AF) (17%), prior TIA (13%) or stroke (10%). Eighty-nine (36%) of the 249 participants taking antiplatelet therapy had no known history of cardiovascular morbidity. Of 102 participants with known AF, 91 (89%) had a CHA2DS2-VASc score ≥ 2 but only 47 (46%) were taking anticoagulation therapy. Among 304 participants taking an antiplatelet or anticoagulant agent, 30 (10%) had stopped taking these in the month prior to the index event.

**Conclusion:** This study provides the first contemporary data on TIAMS or TIAMS-mimics in Australia. Community and health provider education is required to address the under-use of anticoagulation therapy in patients with known AF, possibly inappropriate use of antiplatelet therapy and possibly inappropriate discontinuation of antiplatelet or anticoagulation therapy.

## Introduction

Transient ischemic attack and minor stroke (TIAMS) account for ~40% of all cerebral ischemic events (1, 2) and can precede a disabling or fatal stroke (3) and major cardiovascular events (4, 5). Recurrent stroke usually occurs very early after TIAMS (6) and rapid specialist assessment and intervention has been shown to reduce this risk (7, 8). Therefore, a rapid response within the system of care is required to initiate secondary prevention strategies (4, 8). Current evidence-based guidelines include recommendation for rapid treatment and/or rapid specialist referral (9). Clinical diagnosis of TIAMS is often challenging and it can be difficult to distinguish TIAMS from benign, low risk “stroke-mimic” syndromes (10, 11). Further, it is uncertain how compliant primary health care practitioners are with guidelines directed at the management of TIAMS and how applicable the guidelines are in primary care settings.

In the INternational comparison of Systems of care and patient outcomes In minor Stroke and Tia (INSIST) study (12), we recruited “possible-TIAMS” patients presenting to primary and secondary care settings. The aim of this study was to describe baseline patient characteristics, outcomes and systems of care of all patients enrolled into the INSIST study.

## Methods

**Figure 1:**
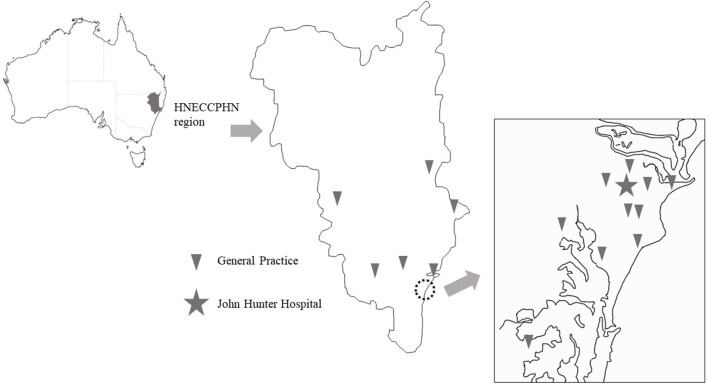
Locations of participating general practices in the Hunter New England and Central Coast Primary Health Network (HNECCPHN) region.

The rationale and methods of the INSIST study have been previously reported (12). Patients were recruited from the Hunter and Manning valley regions of New South Wales, within the referral territory of the Acute Neurovascular Clinic (ANVC) at the John Hunter Hospital between August 2012 and August 2016. The John Hunter Hospital is the major teaching and referral hospital of the Hunter New England Local Health District. Patients attended one of 16 general practices (11 urban and 5 rural practices including 2 where general practitioners (GPs) staff the local hospitals) within the geographic footprint of the Hunter New England and Central Coast Primary Health Network (HNECCPHN) (Figure 1). The estimated population within the HNECCPHN region is 1,200,000 of whom 18.3% are aged 65+ years (compared to 14.4% nationally) and 18.5% have circulatory system disease (17.3% prevalence nationally) (13).

In this study, TIAMS was ascertained using multiple overlapping methods; primarily general practice records but also from hospital emergency departments, after-hours GP services, hospital inpatient admissions and discharges and ANVC attendance at John Hunter Hospital (12). All patients with a possible diagnosis of TIAMS were invited to participate. Episodes with symptoms lasting <24 h were classified as TIAs, and prolonged (>24 h) episodes with National Institute of Health Stroke Scale < 5 at presentation were classified as minor strokes. Patients with both (i) symptoms lasting more than 24 h and (ii) National Institute of Health Stroke Scale >5 at presentation, were excluded. Information recorded at the baseline interview included demographics (age, sex, ethnicity, residence, marital status, education, pre-event employment status, recent occupation, socioeconomic status of area residence [Socio-Economic Index for Area, Index of Relative Socio-economic Disadvantage [SEIFA IRSD] (14)], lifestyle factors (smoking status, alcohol consumption, exercise hours), past medical history and pre-event medications. Further data was obtained from participants' clinical notes.

## Results

During this study, there were 1,363 patients where TIAMS was considered a possible diagnosis whereby 643 consented to participate (response rate 47%), and 613 (335 women; mean [SD] age, 69.8 [12.0] years) completed the baseline interview. Among 613 participants, 298 were adjudicated as TIAMS of whom 175 (29%) had TIA and 123 (20%) had minor stroke. Prior to the index event, 550 (90%) participants were functionally independent (Modified Rankin Scale 0–2).

**Figure 2:**
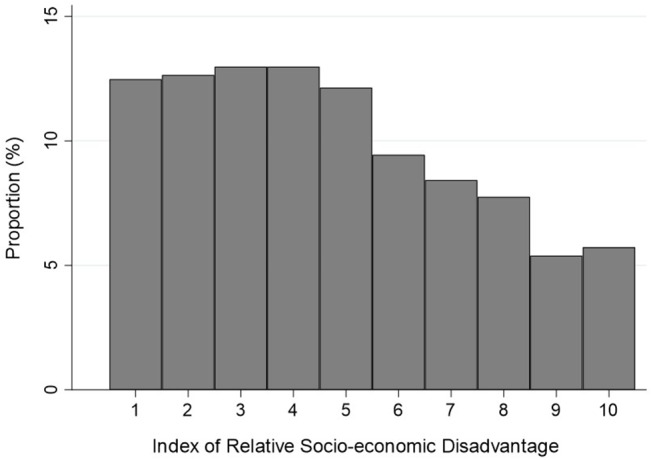
Index of Relative Socio-economic Disadvantage. This index is a general socio-economic index that summarizes a wide range of information about the economic and social resources of people and households within an area. Because this index focuses on disadvantage, only measures of relative disadvantage are included. This means that a high score reflects a relative lack of disadvantage rather than relative advantage.

Participants were somewhat older (on average, by 3 years), and more frequently had focal neurological symptoms, compared to non-participants (Supplemental Material). Most patients were represented across three age bands; 65–74 years (209, 34%), 75–84 years (182, 30%) and 55–64 years (106, 17%) (Table 1). Almost all the participants (604, 99%) were Caucasian. There were differences between male and female participants in marital status and living arrangements with women more likely to be widowed (77, 23% of women vs. 28, 10% of men), living alone (100, 30% vs. 48, 17%) and living in rental accommodation (39, 12 vs. 24, 9%) (Table 2). Education status was divided into more or less than 10 years of schooling with 365 (60%) having education beyond 10 years (Table 3). Attainment of high school certificate was the most common education level of men (92, 33%) and women (81, 24%). Most of the participants (433, 71%) were retired (330, 54% on a government pension and 103, 17% on a self-funded retirement) and 136, 22% were working (70, 11% in full time and 66, 11% in part time/casual employment). The distribution of socioeconomic status of area (SEIFA IRSD—an area-based weighted measure of social and economic resources of people and household, expressed as a decile) is shown in Figure 2.

**Table 1:** Age and sex structure of the study populations.

**Age band**	**Total *n*, (%)**	**Men *n*, (%)**	**Women *n*, (%)**
<45	19 (3.1)	3 (1.0)	16 (4.8)
45–54	44 (7.2)	19 (6.8)	25 (7.5)
55–64	106 (17)	51 (18)	55 (16)
65–74	209 (34)	106 (38)	103 (31)
75–84	182 (30)	73 (26)	109 (33)
≥85	53 (8.6)	26 (9.4)	27 (8.1)
Total	613	278	335

**Table 2:** Ethnicity, residence, housemate, and marital status in enrolled patients.

	**Total, *N* = 613, (%)**	**Men, *N* = 278, (%)**	**Women, *N* = 335, (%)**
**Ethnicity**			
Caucasian	604 (99)	273 (98)	331 (99)
Australian	496 (81)	222 (80)	274 (82)
British & Irish	69 (11)	32 (12)	37 (11)
Southern & Eastern European	17 (2.8)	6 (2.1)	11 (3.2)
Northern & Western European	11 (1.7)	9 (3.2)	2 (0.6)
New Zealander	8 (1.3)	3 (1.1)	5 (1.5)
American	3 (0.5)	1 (0.4)	2 (0.6)
Sub-Saharan Africa	2 (0.3)	2 (0.7)	0
Aboriginal	2 (0.3)	1 (0.4)	1 (0.3)
Maori	1 (0.2)	1 (0.4)	0
Polynesian	1 (0.2)	1 (0.4)	0
South East Asian	1 (0.2)	0	1 (0.3)
Southern & Central Asian	1 (0.2)	0	1 (0.3)
North African & Middle Eastern	1 (0.2)	1 (0.4)	0
**Residence**			
Own home	495 (81)	230 (83)	265 (79)
Rented	63 (10)	24 (8.6)	39 (12)
Retirement village or similar	45 (7.3)	20 (7.2)	25 (7.5)
Others	10 (1.6)	4 (1.4)	6 (1.8)
**Living with**			
Partner or family	457 (75)	228 (82)	229 (68)
Alone	148 (24)	48 (17)	100 (30)
Others	8 (1.3)	2 (0.7)	6 (1.8)
**Marital**			
Married/Partner/Defacto	427 (70)	223 (80)	204 (61)
Widowed	105 (17)	28 (10)	77 (23)
Seperated/Divorced	44 (7.1)	16 (6.0)	28 (8.4)
Single	37 (6.0)	11 (4.0)	26 (7.8)

*TIA, transient ischemic attack*.

**Table 3:** Education, pre-event employment and recent occupation in enrolled patients.

	**Total, *N* = 613, (%)**	**Men, *N* = 278, (%)**	**Women, *N* = 335, (%)**
**Education**			
Schooling <10 years	248 (40)	84 (30)	164 (49)
more than 10 years	365 (60)	194 (70)	171 (51)
Postgraduate degree	28 (4.6)	19 (6.8)	9 (2.7)
Graduate Certificate or Diploma	14 (2.3)	6 (2.2)	8 (2.4)
Bachelor Degree	56 (9.1)	29 (10)	27 (8.1)
Advanced Diploma & Diploma	62 (10)	31 (11)	31 (9.3)
Certificate level	173 (28)	92 (33)	81 (24)
School yr 11 and 12	32 (5.2)	17 (6.1)	15 (4.5)
**Pre-event employment status**			
Retired	433 (71)	198 (71)	235 (75)
On government pension	330 (54)	138 (50)	192 (57)
Self-funded	103 (17)	60 (22)	43 (13)
Working	136 (22)	64 (23)	72 (23)
Full time	70 (11)	44 (16)	26 (7.8)
Part time/casual	66 (11)	20 (7.2)	46 (14)
Unable to work and on pension	22 (3.6)	12 (4.3)	10 (3.0)
Others	22 (3.6)	4 (1.4)	18 (5.7)
**Recent occupation**			
Clerical and Administrator	103 (17)	17 (6.1)	86 (26)
Managerial	96 (16)	75 (27)	21 (6.3)
Professional	95 (15)	46 (17)	49 (15)
Laborers	67 (11)	26 (9.4)	41 (12)
Community Personal Service	59 (10)	11 (4.0)	48 (14)
Technicians trades	55 (9.0)	50 (18)	5 (1.5)
Sales	51 (8.3)	9 (3.2)	42 (13)
Machinery operator and drivers	43 (7.0)	37 (13)	6 (1.8)
Others	44 (7.2)	7 (2.5)	37 (12)
Carer prior to the index event	67 (11)	20 (7.2)	47 (14)

In terms of vascular risk factors, 326 (53%) had never smoked tobacco, 251 (41%) were ex-smokers and 36 (5.9%) were current smokers (Table 4). The median age at which the ex-smokers stopped was 40 years for both men and women. Alcohol intake ranged from 0 standard drink equivalents per week in 42% (79, 28% men and 180, 54% women), to 1–5 standard drink equivalents per week in 24% (61, 22% men and 88, 26% women). Ten percent of the population reported no exercise (22, 8% men and 42, 13% women).

**Table 4:** Exercise, Alcohol, and Smoking status in enrolled patients.

	**Total, *N* = 613**	**Men, *N* = 278**	**Women, *N* = 335**
Exercise, hours per week	4 [2–8]	5.5 [3–10]	4 [2–7]
Alcohol, standard drink equivalent per week	1.6 [0–8.9]	5.6 [0–15]	0 [0–4]
**Smoking status**			
Never	326 (53)	111 (40)	215 (64)
Ex	251 (47)	149 (54)	102 (30)
Current	36 (5.9)	18 (6.7)	18 (5.4)
**Per day smokes**			
Current	16 [10–23]	18 [10–24]	15 [10–20]
Ex	15 [5–20]	20 [10–20]	10 [4.3–20]
**Smoking years**			
Current	44 [34–53]	40 [30–45]	48 [40–58]
Ex	20 [10–40]	17 [10–30]	17 [6.3–30]
Age stopped smoking (Ex)	40 [29–60]	40 [27–50]	40 [28–55]
Stopped last 12M, % of Ex	8 (3.2)	3 (2.0)	5 (5.0)

*Data are presented as median [interquartile range] or absolute (percentage) values*.

Hypertension was the most common (425, 69%) risk factor, followed by hyperlipidemia (321, 52%), diabetes mellitus (106, 17%), atrial fibrillation (AF) (102, 17%), previous TIA (78, 13%) and previous stroke (64, 10%) (Table 5). At the time of the possible TIAMS, 249 (40%) participants were taking antiplatelet therapy (215, 35% single and 34, 5.5% dual antiplatelet therapy) with aspirin being the most common single agent (176, 29% of all participants) (Table 6).

**Table 5:** Past medical histories in enrolled patients.

	**Total, *N* = 613, (%)**	**Men, *N* = 278, (%)**	**Women, *N* = 335, (%)**
Previous stroke	64 (10)	32 (12)	32 (9.6)
Previous TIA	78 (13)	48 (17)	30 (9.0)
Previous funny turn^*^	149 (24)	55 (20)	94 (28)
Hypertension	425 (69)	197 (71)	228 (68)
Diabetes mellitus	106 (17)	60 (22)	46 (14)
Hyperlipidemia	321 (52)	135 (49)	186 (56)
Atrial fibrillation	102 (17)	52 (19)	50 (15)
Angina	101 (16)	58 (21)	43 (13)
Previous myocardial infarction	73 (12)	43 (15)	30 (9.0)
Heart failure	51 (8.3)	20 (7.2)	31 (9.3)
CABG or Angioplasty	71 (12)	50 (18)	21 (6.3)
Carotid endarterectomy	17 (2.8)	15 (5.4)	2 (0.6)
Peripheral vascular disease	40 (6.5)	28 (10)	12 (3.6)
Cancer	125 (20)	65 (23)	60 (18)
Migraine	200 (33)	52 (19)	148 (44)
Epilepsy	16 (2.6)	9 (3.2)	7 (2.1)

* *Funny turn is a non-specific diagnosis (potentially includes neurological, cardiovascular, metabolic, vestibular, and psychological conditions)*.

*CABG, coronary artery bypass grafting; TIA, transient ischemic attack*.

**Table 6:** Pre-event medications in enrolled patients.

	**Total, *N* = 613, (%)**	**Men, *N* = 278, (%)**	**Women, *N* = 335, (%)**
Antiplatelet	249 (40)	130 (47)	119 (36)
Single	215 (35)	109 (39)	106 (32)
Aspirin	176 (29)	93 (33)	83 (25)
Clopidogrel	39 (6.3)	16 (5.8)	23 (6.9)
Dual	34 (5.5)	21 (7.6)	13 (3.9)
Aspirin + Clopidogrel	17 (2.8)	10 (3.6)	7 (2.1)
Aspirin + Dipyridamole	17 (2.8)	11 (4.0)	6 (1.8)
Anticoagulant	56 (9.1)	22 (7.9)	34 (10)
Warfarin	45 (7.3)	17 (6.1)	28 (8.4)
NOAC	11 (1.8)	5 (1.8)	6 (1.8)
Stopped antiplatelet or anticoagulant^*^	30 (4.9)	23 (8.3)	7 (2.1)
Statin	227 (37)	106 (38)	121 (36)
Maximum dose	25 (4.4)	16 (5.8)	9 (2.7)
Antihypertensive drug	394 (64)	184 (66)	210 (63)

* *Stopped in a month prior to the index event*.

*NOAC, non-vitamin K antagonist oral anticoagulants*.

Two hundred and thirty-one participants (38%) had at least one known cardiovascular morbidity (stroke, transient ischemic attack, angina, myocardial infarction, coronary artery bypass grafting or angioplasty, carotid endarterectomy and peripheral vascular disease) at the time of the event, of whom 158 (68%) were taking an antiplatelet agent, 33 (14%) were taking an anticoagulation agent, including 28 with AF. 89 (36%) of the 249 people taking antiplatelet therapy had no known history of cardiovascular morbidity. Among these 89 patients, 19 (21%) had a history of “funny turn” (funny turn is not a specific diagnosis and potentially includes neurological, cardiovascular, metabolic, vestibular and psychological condition), 7 (7.9%) had an AF history and 5 (5.6%) had both history of a funny turn and AF. Of these 12 participants with AF, six had a CHA2DS2-VASc score ≥2.

We found that 56 of 613 (9.1%) participants were taking an anticoagulant agent at the time of the event, 45 (7.3%) warfarin and 11 (1.8%) a non-vitamin K antagonist oral anticoagulant (NOAC). In patients with a known diagnosis of AF, 47 (46%) were taking an anticoagulant despite 91 (89%) having a CHA2DS2-VASc score ≥ 2. Among 304 participants taking an antiplatelet or anticoagulant agent, 30 (10%) had stopped taking in the month prior to the index event. Anti-platelet or anti-coagulant medications were stopped in preparation for an invasive procedure in 14 (47%), by the participant on their own volition in 9 (30%), because the participant forgot to take the medication in 4 (13%), and because of bleeding in 1 (3.3%). 227 (37%) participants were taking a statin (11% at recommended maximum dose) and 394 (64%) were taking antihypertensive agents.

## Discussion

The diagnosis of TIAMS and its management are complex, This study provides a comprehensive and contemporary description of “possible-TIAMS” patients who have engaged with medical care.

### Generalizability of Findings—Ethnicity and Socioeconomic Status

All but nine participants were Caucasian, which is in stark contrast to the situation in Auckland, New Zealand where 78% of TIA patients were of European origin, 11% Maori/Pacific and 10% Asian/Other (15). In our study region, 48,002 (4.2%) identify as Aboriginal and/or Torres Strait Islanders (compared to 2.5% nationally) and a lower proportion of people reported being from non-English speaking backgrounds (4.5% compared to 15.7% nationally) (13). Aboriginal and Torres Strait Islander people have a three-fold greater risk of cerebrovascular diseases, and two-fold neurological conditions risk, compared to non-Indigenous Australians (16). The low proportion of Aboriginal or Torres Strait Islander participants may reflect the fact that none of the Aboriginal Medical Services in the region (where an appreciable proportion of the Aboriginal and Torres Strait Islander community access health care) elected to participate in the study. It is also possible that our findings reflect lower rates of presenting to primary or secondary care with neurological symptoms in this group.

Our study population was of relatively lower socioeconomic status compared to the distribution in the HNECCPHN population (IRSD decile 6 was the most common (42%) followed by decile 7 (32%) reported from the Australian Census 2016) (14). The proportion of participants in each SEIFA IRSD decile declining as socio-economic status decile increased. Low socioeconomic status has been associated with fatal stroke and non-fatal stroke incidence (17). Education is one of the many constituent measures included in the SEIFA– IRSD and we note that 40% of our study population had <10 years of education.

### Baseline Antiplatelet and Anticoagulation Status

About one third of patients were taking antiplatelet therapy (38%) prior to the index event. This likely reflects the prior history of vascular disease. However, many of the participants were taking antiplatelet agents without an apparent indication. This finding of possible inappropriate prescription of antiplatelet agents is consistent with previous studies (18, 19). Anticoagulant therapy was underused considering the CHA2DS2-VASc score in the study population, with 46% of participants with a known diagnosis of AF treated (20). This is consistent with findings of previous studies where 23–30% of TIA patients with known AF were taking anticoagulation therapy at the time of the TIA (15, 21, 22).

This study provides evidence of a clear need for optimization of antiplatelet and anticoagulation therapy in patients with established vascular disease and AF. In particular, GPs should be encouraged to calculate CHA2DS2-VASc scores and consider the need for anticoagulation in patients presenting with AF. Presentation with possible-TIAMS provides a key opportunity to start anticoagulantion therapy if appropriate based on risk stratification and bleeding risk. The finding that antiplatelet or anticoagulation therapy had been stopped in the month prior to the index event in many patients is also a cause for concern. This highlights a need for GPs to carefully consider the potential risks and benefits when considering cessation of anticoagulation or antiplatelet therapy, and the need to promptly reinstitute therapy as soon as it is safe after temporary discontinuation.

### Statin and Antihypertensive Preventive Treatment

The prevalence of statin (37%) and antihypertensive therapy (64%) used in the study population seems modest given prevalence of known hyperlipidaemia (52%) and hypertension (69%), as well as the prevalence of diabetes mellitus (17%) and previous stroke (13%). In an absolute cardiovascular risk (ACVR) analysis of a subgroup participating early in the INSIST study, 72% of participants in which ACVR could be calculated had high or moderate ACVR (23). Considering this risk proportion, the prevalence of statin and antihypertensive therapy seems relatively modest.

### Strengths and Limitations

This study has a number of strengths. It is a comprehensive community-based study of TIAMS patients engaging with health systems at any level that used multiple overlapping case ascertainment methods for possible TIAMS. The study also has a number of limitations. There was a modest response or consent rate of 47%. This rate is typical of declining response rates for cohort studies (24). We did not record a past history of hemorrhage or other potential contraindications to anti-thrombotic therapy. The study was performed at a time when non-vitamin K antagonist oral anticoagulants were still relatively new with subsequent rapid uptake of these medicines and corresponding reductions in the use of warfarin (25, 26). There was a lack of representation of the Aboriginal and Torres Strait Islander community and lower number of culturally and linguistically diverse individuals than would be expected, reflecting the recruitment catchment demographics. We are therefore unable to generalize the findings to non-Caucasian populations.

## Conclusions

This study provides the first contemporary data on TIAMS or TIAMS-mimics in Australia. Worryingly, we found under-use of anticoagulation therapy in patients with known AF prior to the index event and possibly inappropriate use of antiplatelet therapy. Community and health provider education is required to address the under-use of anticoagulation therapy in patients and possibly inappropriate discontinuation of antiplatelet or anticoagulation therapy.

## Data Availability Statement

All datasets generated for this study are included in the article/Supplementary Material.

## Ethics Statement

The studies involving human participants were reviewed and approved by Hunter New England Human Research Ethics Committee (The Reference No. is 12/04/18/4.02). The patients/participants provided their written informed consent to participate in this study.

## Author Contributions

Study design: CL, PM, DL, and DQ. Data collection: DQ, HZ, CG-E, AD, NN, and MS. Data analysis: PM, DQ, and ST. Writing: ST, PM, and CL. Manuscript revision: DL, JV, HD, PB, NS, DC, and VF. All contributed for writing and revision: DL, PB, and PR.

## Conflict of Interest

The authors declare that the research was conducted in the absence of any commercial or financial relationships that could be construed as a potential conflict of interest.
