# Supplementary material for: The Characteristics of Patients With Possible Transient Ischemic Attack and Minor Stroke in the Hunter and Manning Valley Regions, Australia (the INSIST Study)
Source: Front Neurol. 2020 May 15;11:383. doi: 10.3389/fneur.2020.00383 (PMC7326044; doi:10.3389/fneur.2020.00383)
Supplement: Supplementary file 1 [file Table_1.DOCX]

**Supplemental Material**

**Table I.** A comparison of age, gender and clinical symptoms between participants and non-participants

|  | Total, n = 1,243, (%) | Participants, n = 613, (%) | Non-participants*, n = 630, (%) | P value** |
| --- | --- | --- | --- | --- |
| Age, mean, SD | 68.2 ± 14.0 | 69.8 ± 12.0 | 66.7 ± 15.6 | < 0.001 |
| Gender, Female | 682 (55) | 335 (55) | 347 (55) | 0.879 |
| Symptom |  |  |  |  |
| Motor disturbance | 378 (30) | 176 (29) | 202 (32) | 0.199 |
| Sensory disturbance | 314 (25) | 168 (27) | 146 (23) | 0.086 |
| Visual disturbance | 342 (28) | 190 (31) | 152 (24) | 0.007 |
| Monocular | 92 (7.4) | 51 (8.3) | 41 (6.5) | 0.224 |
| Speech disturbance | 362 (29) | 196 (32) | 166 (26) | 0.029 |
| Ataxia | 230 (19) | 172 (28) | 58 (9.2) | < 0.001 |
| More than one of the above symptoms | 519 (42) | 291 (48) | 228 (36) | < 0.001 |
| None of the above symptoms | 245 (20) | 84 (14) | 161 (26) | < 0.001 |

*Among 720 patients who did not consent to participate, 90 did not meet the criteria and excluded.

**Chi square test was performed in STATA 15.1 (Stata Corp, College Station, Texas).
